# Antibiotics influence the toxicity of the delta endotoxins of *Bacillus thuringiensis* towards the cotton bollworm, *Helicoverpa armigera*

**DOI:** 10.1186/1471-2180-14-200

**Published:** 2014-07-24

**Authors:** Inakarla Paramasiva, Hari C Sharma, Pulipaka Venkata Krishnayya

**Affiliations:** 1 International Crops Research Institute for the Semi-Arid Tropics (ICRISAT), Patancheru 502 324 Andhra Pradesh, India; 2 Acharya N G Ranga Agricultural University, Agricultural College, Bapatla, Andhra Pradesh, India

## Abstract

**Background:**

The cotton bollworm, *Helicoverpa armigera* is one of the most important crop pests worldwide. It has developed high levels of resistance to synthetic insecticides, and hence, *Bacillus thuringiensis* (*Bt*) formulations are used as a safer pesticide and the *Bt* genes have been deployed in transgenic crops for controlling this pest. There is an apprehension that *H. armigera* might develop resistance to transgenic crops in future. Therefore, we studied the role of gut microbes by eliminating them with antibiotics in *H. armigera* larvae on the toxicity of *Bt* toxins against this pest*.*

**Results:**

Commercial formulation of *Bt* (Biolep®) and the pure Cry1Ab and Cry1Ac toxin proteins were evaluated at ED_50_, LC_50_, and LC_90_ dosages against the *H. armigera* larvae with and without antibiotics (which removed the gut microbes). Lowest *H. armigera* larval mortality due to *Bt* formulation and the *Bt* toxins Cry1Ab and Cry1Ac was recorded in insects reared on diets with 250 and 500 μg ml^−1^ diet of each of the four antibiotics (gentamicin, penicillin, rifampicin, and streptomycin), while the highest larval mortality was recorded in insects reared on diets without the antibiotics. Mortality of *H. armigera* larvae fed on diets with *Bt* formulation and the δ-endotoxins Cry1Ab and Cry1Ac was inversely proportional to the concentration of antibiotics in the artificial diet. Nearly 30% reduction in larval mortality was observed in *H. armigera* larvae from F_1_ to F_3_ generation when the larvae were reared on diets without antibiotics (with gut microbes) and fed on 0.15% *Bt* or 12 μg Cry1Ab or Cry1Ac ml^−1^ diet, indicating development of resistance to *Bt* in the presence of gut microflora. However, there were no differences in larval mortality due to *Bt*, Cry1Ab or Cry1Ac across generations in insects when they were reared on diets with 250 μg of each antibiotic ml^−1^ diet (without gut microflora).

**Conclusions:**

The results suggested that antibiotics which eliminated gut microflora influenced the toxicity of *Bt* towards *H. armigera*, and any variation in diversity and abundance of gut microflora will have a major bearing on development of resistance to *Bt* toxins applied as foliar sprays or deployed in transgenic crops for pest management.

## Background

The cotton bollworm, *Helicoverpa armigera* (Hubner) (Lepidoptera: Noctuidae) is the most damaging pest of cotton, grain legumes, cereals, vegetables, fruit crops, and forest trees in Asia, Africa, Australia, and the Mediterranean Europe. It is a polyphagous insect that causes an estimated loss of US$2 billion in different crops worldwide [1]. *Helicoverpa armigera* control is currently heavily based on insecticide use, and it has developed high levels of resistance to commonly used insecticides [2]. To overcome this problem, transgenic crops expressing *Bacillus thuringiensis* (*Bt*) (Berliner) toxin proteins have been deployed on a large scale for pest management worldwide, that has resulted in a considerable reduction in pesticide use and increased crop production and profitability [3].

*Bacillus thuringiensis* is a spore forming bacterium that produces crystal proteins (Cry proteins), which are toxic to many species of insects [4,5]. In Lepidoptera, specificity to *Bt* Cry toxins is due to alkaline pH of the mid gut, which is essential for conversion of protoxin to active toxin form [6-8]. However, increasing use of *Bt* as a conventional insecticide and large-scale cultivation of *Bt-*transgenic crops may lead to evolution of resistance in *H. armigera* populations to *Bt* toxins. Therefore, there is a need to develop strategies to manage development of resistance to *Bt* toxins, taking into account the various resistance and/or detoxification mechanisms involved in the target and non-target insect pests in different eco-systems.

Development of resistance to *Bt* toxins has largely been attributed to the extent of binding of *Bt* toxin proteins to brush border membrane vesicles. However, the toxicity of *Bt* toxins to insects also depends on the enteric bacteria such as *Escherichia coli* (Migula) and *Enterobacter* sp. that normally reside in the insect mid gut [9]. There is considerable variation in the susceptibility of *H. armigera* larvae to *Bt* toxins from different locations, and from different host plants [10], which may be due to variation in gut microflora involved in insect nutrition, and potentiation/degradation of *Bt* toxin proteins in the insect mid gut. Therefore, diversity and population intensity of the enteric bacteria in the mid gut will have a bearing on the insect susceptibility to *Bt* toxins deployed in transgenic crops for pest management.

Interactions of gut microflora with infectious pathogens and toxins of pathogen or toxins of plant origin have been studied in many organisms, including Crustacea, Mollusca, Echinodermata [11], gypsy moth, butterflies [12], and rats [13]. The crystal proteins of *B. thuringiensis* var *alesti* have shown little lethality to cabbage looper larvae, *Trichoplusia ni* (Hub.) reared on diets containing chlortetracycline at a concentration of 130 ppm [14]. Addition of chlortetracycline hydrochloride in the artificial diet decreased the biological activity of HD-1 strain of *B. thuringiensis* by 2.4 – 67.1 times against 4-day-old larvae of *T. ni, Heliothis virescens* (F.), and *Ostrinia nubilalis* (Hub.) [15]. Presence of either bacterial spores or vegetative cells of *E. coli* several forest epiphytic bacteria significantly increased the biological activity of *Bt* toxins CryIAa and CryIAc against the gypsy moth, *Lymantria dispar* (L.) [16]. *Bacillus thuringiensis* resulted in 63 to100% mortality in *Vanesa cardui* L.*, Manduca sexta* (L.)*, Pieris rapae* (L.)*,* and *H. virescens* reared on diets without antibiotics, but only 0 to 10% mortality was recorded when the larvae were reared on diets amended with antibiotics [17]. Antibiotics reduced the populations of gut bacteria below detectable limits, except in *H. virescens*, which had detectable bacteria prior to treatment with *Bt* toxins. The reduction in insect mortality was associated with reduced populations of *Enterococcus* and *Enterobactor* species from the mid guts of the larvae [9]. Re-establishment of *Enterobactor* sp., which resides in the insect mid gut, restored the toxicity of *Bt* toxins [9,17]. However, subsequent studies suggested that reduction in *Bt* toxicity was not due to removal of gut bacteria, but due the effect of the antibiotics [18-20]. However, some of these studies used a different methodology, wherein, the antibiotics aureomycin and ampicillin were administered post *Bt* treatment. The aseptic larvae that were exposed to the antibiotics during the bioassay or continuously exposed to the antibiotics died more slowly when treated with *Bt*. However, the larvae administered with a cocktail of antibiotics conferred resistance to *L. dispar* against *Bt*[20]. *Bacillus cereus* (Fr. & Fr.) strains secreting antibiotics synergised *Bt* infection in the diamond back moth, *Plutella xylostella* L*.*, but at levels lower than reported earlier [21]. Ingestion of antibiotic secreting strains reduced the abundance of gut microflora regardless of the genotype, reduced the densities of enteric isolates of the gut bacterium, *Enterobactor* sp., suggesting that secretion of antibiotics in the mid gut synergises the *Bt* infection by reducing the abundance of commensal gut microflora.

Elimination of gut microbial community by oral administration of antibiotics significantly decreased the insecticidal activity of *Bt*, and re-establishment of an *Enterobacter* sp., that normally resides in the mid gut, restored the *Bt*-mediated toxicity. However, *B. thuringiensis* is unable to grow in the insect hemocoel [22,23], suggesting that other microbes residing the insect gut play an important role in biological activity of *Bt* toxins against different insect pests. Because of the profound effect of antibiotics through elimination of gut microbes on the toxicity of *Bt* toxin proteins, it is important to study these interactions to understand the underlying mechanisms of potentiation and/or degradation of Cry toxins by gut microflora to unravel the possible evolution of various mechanisms of resistance to *Bt* used as pesticide sprays or deployed in transgenic crops for pest management.

## Results

### Effect of different concentrations of antibiotics on the mortality of *H. armigera* larvae due to *Bt* toxins

#### *Bt* formulation

**Figure 1:**
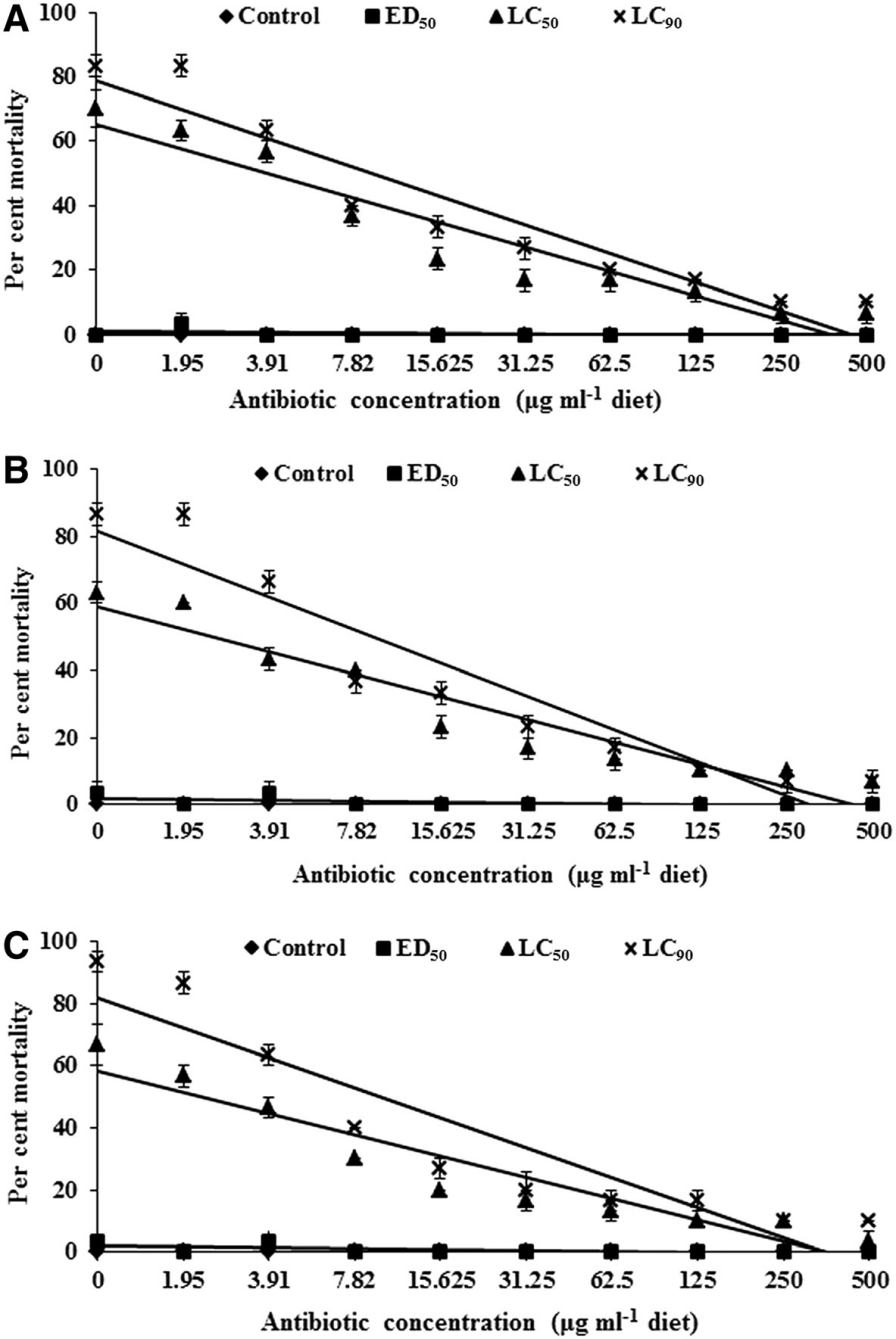
**Effect of antibiotics in the artificial diet on mortality of *Helicoverpa armigera *** **larvae due to *Bt*-formulation (A), and the toxin proteins Cry1Ab (B) and Cry1Ac (C).**

The mortality of *H. armigera* larvae due to *Bt* at LC_50_ (0.15%) (Y = 72.67 – 7.58x, R^2^ = 90.65%; where Y = larval mortality, x = *Bt* concentration, and R^2^ = coefficient of determination, i.e. percentage variation in larval mortality explained by antibiotic concentrations) and LC_90_ (0.95%) (Y = 87.77 - 8.93x , R^2^ = 90.74%) concentrations decreased with an increase in the dosage of antibiotics from 1.95 to 500 μg ml^−1^ (Figure 1A). The *Bt* formulation at the ED_50_ levels did not cause any larval mortality. There were no significant differences in mortality of *H. armigera* larvae reared on diets with different amounts of the antibiotic mixture without *Bt*, suggesting that antibiotics did not result in any adverse effects on the insect larvae*. Bt f*ormulation at LC_90_ level resulted in 10.00% larval mortality in insects reared on diets with 250 and 500 μg of each antibiotic ml^−1^ diet as compared to 83.33% mortality in larvae reared on diets without antibiotics, suggesting that elimination of the gut microflora by antibiotics decreased the toxicity of *Bt* towards the larvae of *H. armigera.*

#### Cry1Ab

The *H. armigera* larval mortality due to Cry1Ab toxin at the LC_50_ (12 μg ml^−1^ diet) (Y = 65.78 – 6.75x, R^2^ = 91.26%) and LC_90_ (35 μg ml^−1^ diet) (Y = 91.78 - 9.89x, R^2^ = 89.87%) concentrations decreased with an increase in the dosage of antibiotics from 1.95 to 500 μg ml^−1^ (Figure 1B). Cry1Ab at the ED_50_ level did not cause any larval mortality. There were no significant differences in the mortality of *H. armigera* larvae reared on diets with different amounts of the antibiotic mixture, but without Cry1Ab. Highest mortality of 86.67% was observed in *H. armigera* larvae reared on diets with LC_90_ level of the Cry1Ab toxin, but without antibiotics, whereas 6.67% larval mortality was recorded when the insects were reared on diets with 250 and 500 μg of each antibiotic ml^−1^ diet with 35 μg ml^−1^ diet of Cry 1Ab toxin. The antibiotics decreased the activity of Cry1Ab by eliminating the gut microflora that possibly secreted enzymes involved in potentiation/detoxification of *Bt* toxins.

#### Cry1Ac

The mortality of *H. armigera* larvae due to Cry1Ac toxin at LC_50_ (12 μg ml^−1^ diet) (Y = 65.12 – 6.87x, R^2^ = 89.91%) and LC_90_ (35 μg ml^−1^ diet) (Y = 91.55 - 9.67x, R^2^= 85.78%) concentration decreased with an increase in the dosage of antibiotics from 1.95 to 500 μg ml^−1^ diet of each antibiotic (Figure 1C). Larval mortality was 3.33 to 10.00% in insects reared on diets with 4 μg Cry1Ac ml^−1^ diet + antibiotics, but without Cry1Ac. Highest larval mortality (93.33%) was recorded in insects reared on diets with 35 μg Cry1Ac ml^−1^ diet without antibiotics as compared to 10% mortality in larvae reared on diets with 250 and 500 μg of each antibiotic ml^−1^ diet + 35 μg of Cry1 Ac ml^−1^ diet.

### Effect of different concentrations of antibiotics on weight gain by the larvae of *H. armigera* in artificial diets amended with *Bt* toxins

#### *Bt* formulation

**Figure 2:**
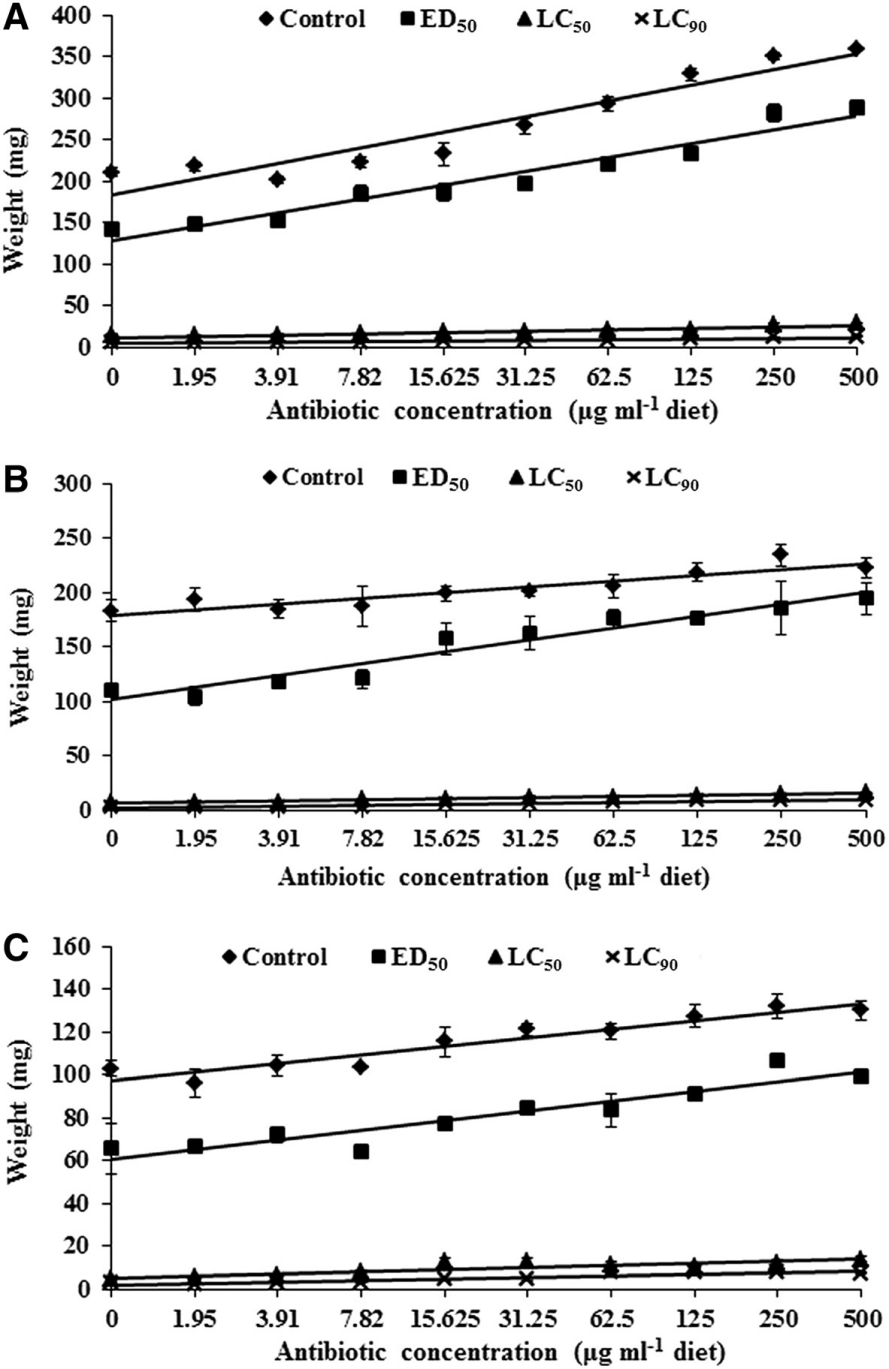
**Effect of antibiotics in the artificial diet on the weights of *Helicoverpa armigera *** **larvae due to *Bt*-formulation (A), and the toxin proteins Cry1Ab (B) and Cry1Ac (C).**

The weights of the *H. armigera* larvae increased with an increase in the antibiotic concentration, but without *Bt* (Y = 163.08 + 19.02x, R^2^ = 90.98%). Similar increase in larval weights was observed in *H. armigera* larvae reared on diets with *Bt* at the ED_50_ level (Y = 111.07 + 16.75x, R^2^ = 94.73%) (Figure 2A). The weights of the *H. armigera* larvae also increased with an increase in the amounts of the antibiotics in the artificial diets + *Bt* at the LC_50_ level, although the larval weights were quite low (Y = 10.01 + 1.62x, R^2^ = 86.11%). Similar trends in increase in larval weights were also observed in insects reared on diets with *Bt* at the LC_90_ level along with antibiotics. However, the larval weights were quite low. The larval weights were lowest (5.71 mg per larva) in insects reared on diets without antibiotics + 0.95% *Bt*, while maximum larval weights (358.61 mg per larva) were recorded in insects reared on diets with 500 μg of each antibiotic ml^−1^ diet, but without *Bt,* suggesting that antibiotics decreased the biological activity of *Bt* formulation against the larvae of *H. armigera*.

#### Cry1Ab

The weights of the *H. armigera* larvae increased with an increase in the antibiotic concentration in the artificial diet without Cry1Ab in the artificial diet (Y = 173.91 + 5.22x, R^2^ = 83.81%) (Figure 2B). Similar increase in weights of *H. armigera* larvae was also observed in insects reared on diets with Cry1Ab at the ED_50_ level (4 μg ml^−1^ diet) (Y = 90.49 + 10.89x, R^2^ = 93.53%). The weights of the *H. armigera* larvae also increased with an increase in the amounts of the antibiotics in diets + Cry1Ab at 12 μg ml^−1^ diet, but the larval weights were quite low (Y = 5.25 + 0.99x, R^2^ = 94.40%). The *H. armigera* larvae weighed only 1.70 mg per larvae when reared on diets with 1.95 μg of each antibiotic ml^−1^ diet + 35 μg Cry1Ab ml^−1^ diet as compared to 234.3 mg per larva in insects reared on diets with 250 μg of each antibiotic ml^−1^, but without Cry1Ab.

#### Cry1Ac

The weights of the *H. armigera* larvae increased with an increase in the antibiotic concentration without Cry1Ac in the artificial diet (Y = 93.06 + 4.05x, R^2^ = 90.51%) (Figure 2C). Similar increase in weights of *H. armigera* larvae was also observed in insects reared on diets with Cry1Ac at the ED_50_ level (4 μg ml^−1^ diet) (Y = 56.58 + 4.78x, R^2^ = 87.21%). The weights of the *H. armigera* larvae also increased with an increase in the amounts of the antibiotics in the artificial diets having Cry1Ac at 12 μg ml^−1^ diet, but the rate of increase was quite low (Y = 4.36 + 1.01x, R^2^ = 78.46%). The larval weights were significantly lower (2.66 mg) in insects reared on diets with 1.95 μg of each antibiotic ml^−1^ + 35 μg Cry1Ac ml^−1^, and on diets without antibiotics + 35 μg Cry1Ac ml^−1^ (2.82 mg) as compared to 132.03 mg in the larvae reared on diets with 250 μg of each antibiotic ml^−1^ diet, but without Cry1Ac.

### Effect of antibiotics on the mortality of *H. armigera* larvae due to *Bt* toxins across three generations

#### *Bt* formulation

**Figure 3:**
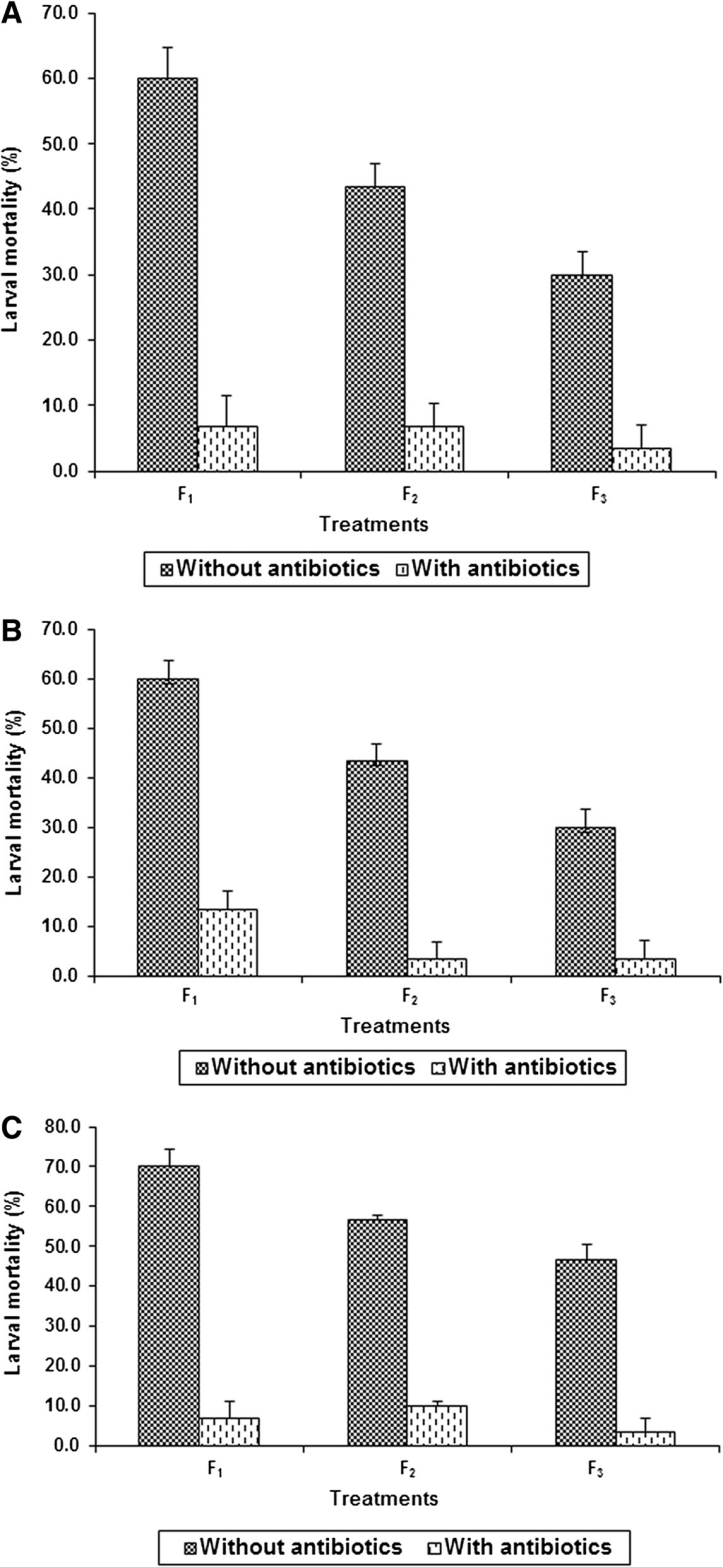
**Effect of antibiotics (250 μg each of gentamicin, penicillin, rifampicin, and streptomycin ml**^**−1 **^**diet) on mortality of *Helicoverpa armigera *larvae with LC**_**50 **_**concentration of *Bt*-formulation (A), and the toxin proteins Cry1Ab (B) and Cry1Ac (C).** LC_50_ = Effective dose to cause 50% larval mortality. F_1_, F_2_, and F_3_ = *Helicoverpa armigera* generations exposed to antibiotics.

Addition of the antibiotic mixture to the artificial diet significantly reduced the mortality of *H. armigera* larvae due to *Bt* formulation in F_1_ generation (*Bt:* Fp = 53.3**, antibiotics: Fp = 29.3**), F_2_ generation (*Bt:* Fp = 73.0***; antibiotics: Fp = 21.2**), and the F_3_ generation (*Bt:* Fp = 41.0**; antibiotics: Fp = 19.3**) (*, ** F-test significant at P 0.05 and 0.01, respectively) (Figure 3A). The interaction effects were also significant in F_1_ (Fp = 18.9**), F_2_ (Fp = 21.3**), and F_3_ (Fp = 19.4**) generations. The larval mortality varied from 3.33 to 6.67% through F_1_ to F_3_ generation when the *H. armigera* larvae were reared on diets with 250 μg antibiotics + 0.15% *Bt* as compared from 60.00% mortality in F_1_ generation and 30.00% mortality in the F_3_ generation in larvae reared on diets without antibiotics + 0.15% *Bt,* suggesting that gut microflora influenced the susceptibility of *H. armigera* to *Bt* toxins across generations.

#### Cry1Ab

Addition of antibiotics to the artificial diet resulted in significant reduction in mortality of *H. armigera* larvae due to Cry1Ab in F_1_ (*Bt:* Fp = 123.9**; antibiotics: Fp = 33.3**), F_2_ (*Bt:* Fp = 60.9**; antibiotics: Fp = 33.3**), and F_3_ (*Bt:* Fp = 41.0**; antibiotics: Fp = 19.4**) generations (Figure 3B). The interaction effects were also significant in F_1_ (Fp = 19.1**), F_2_ (Fp = 33.3**) and F_3_ (Fp = 19.4**) generations. The larval mortality decreased from 13.33% in the F_1_ generation to 3.33% in F_3_ generation in larvae reared on diets with 250 μg of each antibiotic ml^−1^ diet +12 μg Cry1Ab ml^−1^ as compared to 60% mortality in F_1_ to 30% mortality in F_3_ generation in larvae reared on diets without antibiotics + 12 μg Cry1Ab ml^−1^.

#### Cry1Ac

There were significant differences in *H. armigera* larval mortality due to Cry1Ac on diets with and without antibiotics in F_1_ (*Bt:* Fp = 81.3**; antibiotics: Fp = 33.8**), F_2_ (*Bt:* Fp = 1223.9**; antibiotics: Fp = 249.9**), and F_3_ (*Bt:* Fp = 61.9**; antibiotics: Fp = 34.9**) generations (Figure 3C). The interaction effects were significant in F_1_ (Fp = 33.8**), F_2_ (Fp = 249.9**), and F_3_ (Fp = 34.8**) generations. The larval mortality varied from 3.33 to 6.67% in F_1_ to F_3_ generations when the larvae were reared on diets with 250 μg of each antibiotic ml^−1^ + 12 μg Cry1Ac ml^−1^ diet as compared to the 70.00% mortality in F_1_ and 46.67% mortality in F_3_ generation when the larvae were reared on diets without antibiotics + 12 μg Cry1Ac ml^−1^ diet.

### Effect of antibiotics on weight gain by *H. armigera* larvae on diets with and without *Bt* toxins across three generations

#### *Bt* formulation

**Figure 4:**
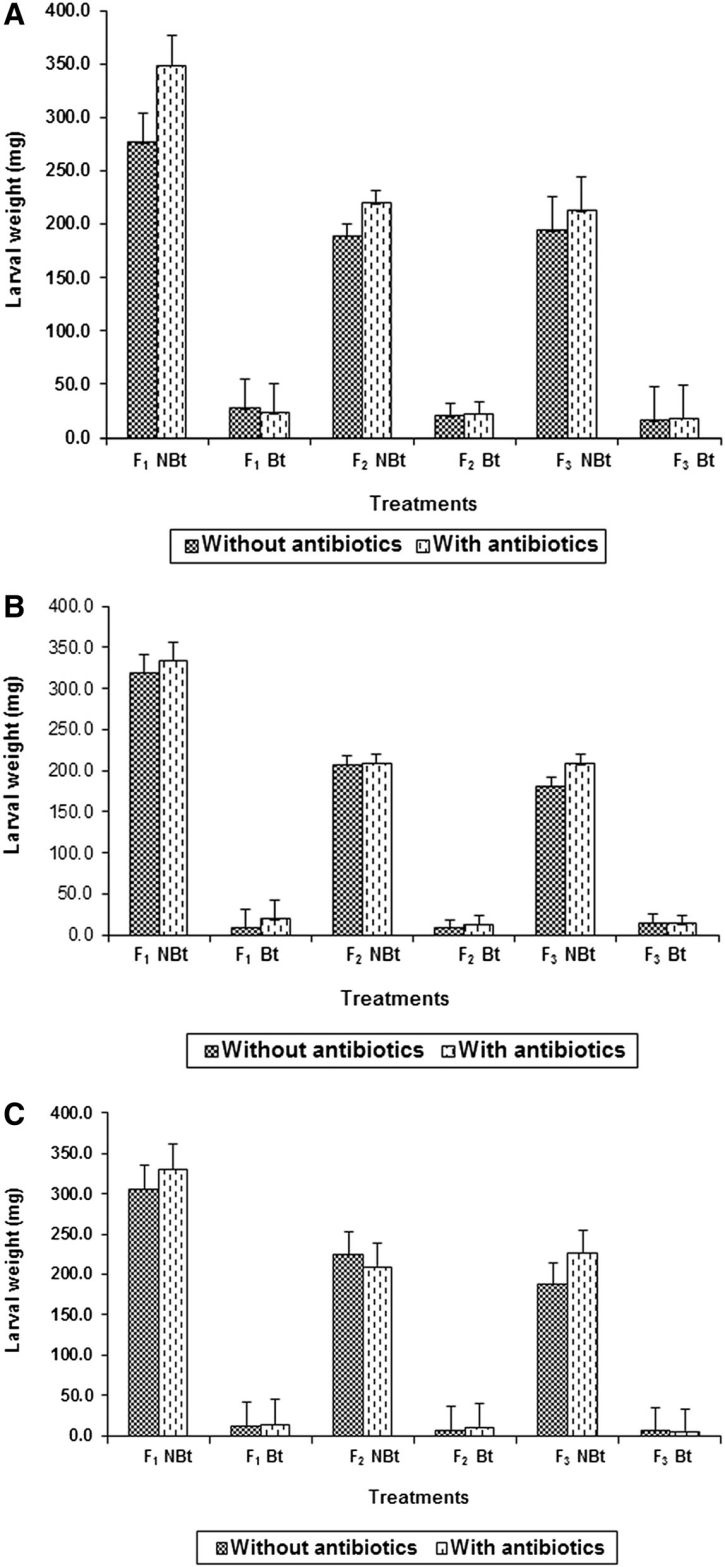
**Effect of antibiotics (250 μg each of gentamicin, penicillin, rifampicin, and streptomycin ml**^**−1 **^**diet) on weights of *Helicoverpa armigera *larvae with LC**_**50 **_**concentration of *Bt*-formulation (A), and toxin proteins Cry1Ab (B) and cry1Ac (C).** LC_50_ = Effective dose to cause 50% larval mortality. F_1_, F_2_, and F_3_ = *Helicoverpa armigera* generations exposed to antibiotics.

Incorporation of antibiotic mixture into the artificial diet resulted in significant differences in weights of *H. armigera* larvae reared on diets with and without *Bt* in F_1_ (*Bt:* Fp = 122.4*; antibiotics: Fp = 5.2**), F_2_ (*Bt:* Fp = 275.1*; antibiotics: Fp = 8.3**), and F_3_ (*Bt:* Fp = 35.6*; antibiotics: Fp = 0.04) generations (Figure 4A). The interaction effects between *Bt* x antibiotic treatments were non-significant. The larval weights declined from 181.3 mg in F_1_ generation to 112.0 mg in the F_3_ generation when the larvae were reared on diets without antibiotics as compared to 217.8 mg in F_1_ and 122.9 mg in F_3_ generation on diets with antibiotics.

#### Cry1Ab

Addition of antibiotics resulted in significant differences in *H. armigera* larval weights on diets with and without Cry1Ab in F_1_ (Fp = 72.5*), F_2_ (Fp = 130.5*), and F_3_ (Fp = 91.9*) generations, but the antibiotic and *Bt* interaction effects were non-significant (Figure 4B). The larval weights decreased from 190.62 mg in F_1_ generation to 106.80 mg in the F_3_ generation in the larvae reared on diets without antibiotics as compared to 206.3 mg in F_1_ and 115.3 mg in F_3_ generation in larvae reared on diets with antibiotics.

#### Cry1Ac

The larval weights differed significantly in larvae of *H. armigera* reared on diets with Cry1Ac, with and without antibiotics in F_1_ (*Bt:* Fp = 101.9**), F_2_ (*Bt:* Fp = 52.6*), and F_3_ (*Bt:* Fp = 52.2*) generations (Figure 4C). The interaction effects between Cry1Ac x antibiotics were non-significant. There was a significant reduction in larval weights from 164.4 mg in F_1_ generation to 94.1 mg in F_3_ generation in larvae reared on diets without antibiotics as compared to 182.4 mg in F_1_ and 121.2 mg in F_3_ generation in larvae reared on diets with antibiotics.

## Discussion

Microorganisms play an important role in physiology and nutrition of insects [24-27], and also provide protection against infectious pathogens and toxins of pathogen or plants in many organisms [11-13]. In the present studies, we studied the role of gut microbes in *H. armigera* on the biological activity of *Bt* toxins by using antibiotics to eliminate the gut microflora. Commercial *Bt* formulation and the toxin proteins Cry1Ab and Cry1Ac induced larval mortality was reduced significantly when the larval diet was amended with antibiotics in a dose-dependent manner. Mortality of insects fed with *Bt* toxins was inversely proportional to the antibiotic concentration. Decrease in microbial population with an increase in the dosage of the antibiotics resulted in a proportionate decrease in larval mortality. Lowest larval mortalities were recorded in insects reared on diets with 500 and 250 μg of each antibiotic ml^−1^ diet along with LC_50_ concentrations of *Bt* formulation and Cry toxins, while maximum mortality was recorded in insects reared on diets without antibiotics + LC_90_ concentration of *Bt* formulation/Cry toxins. Mortality of gypsy moth, *L. dispar* and cabbage looper, *T. ni* fed on *Bt* amended diet has also been reported to be inversely proportional to the antibiotic concentration [9,14,28]. Jarosz [29] observed that *Streptococcus faecalis* (Andrewes & Horder) in guts of *G. mellonella* larvae suppressed the bacteria ingested with food by producing bacteriocin - an antibiotic like substance having a narrow range of bactericidal activity, and by releasing a lysozyme like enzyme. The reduction in mortality due to *Bt* was attributed to reduced populations of gut bacteria, *Enterococcus* and *Enterobacter* species. Re-establishment of *Enterobacter* sp. in larvae reared on antibiotic-amended diet restored the biological activity of *Bt*[9].

The growth of *Bt* drops to below detectable limits in the insect hemolymph after 6 h, whereas *Enterobacter* sp. and *E. coli* grow rapidly in larval hemolymph, indicating that the enteric bacteria are needed for septicaemia associated with *Bt* toxicity. *Bt* does not grow in the insect hemocoel, indicating that *Bt* alone is not responsible for septicaemia [23,30]. However, enteric bacteria alone do not induce mortality, suggesting that *Bt* enables them to reach the hemocoel by making the gut epithelium permeable [9]. These findings are further supported by the earlier observations that the *Bt* spores are absent from the insect hemocoel until very late in the infection process, and frequently do not appear until well after the death of the insect [22,31-33] Incorporation of *Pseudomonas* and *Acinetobacter* isolates in the mosquito blood meal result in an increased susceptibility of *Culex quiquefasciatus* Say to the Japanese encephalitis virus [34].

Role of gut microflora in the susceptibility of *H. armigera* larvae to *Bt* was also studied across three generations by rearing the insects on artificial diet amended with antibiotics with and without *Bt* toxins. There was nearly 30% reduction in larval mortality from F_1_ to F_3_ generations when the larvae were reared on diets without antibiotics (with gut microbes) and fed on 0.15% *Bt* formulation, 12 μg Cry1Ab ml^−1^ diet or 12 μg Cry1Ac ml^−1^ diet. However, when the insects were reared on diets with antibiotics (without gut microflora), there was no significant reduction in insect mortality from F_1_ to F_3_ generations.

Antibiotics have been reported to reduce the populations of gut microflora in many insect species and the reduction in insect mortality is associated with reduced populations of gut microflora, including *Enterococcus* and *Enterobactor* species from the mid guts of the larvae [9,14-17]. Re-establishment of *Enterobactor* sp., which resides in the insect mid gut microbial community, restored the toxicity of *Bt* toxins [9,17]. *Bacillus cereus* strains secreting antibiotics also synergise *Bt* infection in the diamond back moth, *P. xylostella*[21]. Ingestion of antibiotic secreting strains reduced the abundance of gut microflora, and regardless of the genotype, reduced the densities of enteric isolates of the gut bacterium, *Enterobactor* sp., suggesting that antibiotic secretion in the gut synergises the *Bt* infection by reducing the abundance of commensal gut microflora. However, some workers have suggested that enteric bacteria did not contribute to insect mortality due to *Bt*[18-20], and the reduction in *Bt* toxicity was due the effect of the antibiotics on toxicity of *Bt* toxins. However, they used a different methodology, wherein, the antibiotics aureomycin and ampicillin were administered post *Bt* treatment. However, the larvae administered with a cocktail of antibiotics conferred resistance to *L. dispar* against *Bt*[20]. The aseptic larvae that were exposed continuously or only during the bioassay died slowly when treated with *Bt*. The weights of *H. armigera* larvae increased with an increase in antibiotic concentration in the diet, indicating that elimination of gut microbes reduced the biological activity of *Bt*, but not the larval growth, suggesting that these enteric bacteria do not cause any adverse effects on *H. armigera* larvae or play a major role in nutrition of *H. armigera*.

From the foregoing account, it is apparent that antibiotics reduce the activity of gut microflora in the insect mid gut, but the extent and the nature of effect on different species varies with the antibiotic used, administration of the antibiotics (before or after treatment with *Bt* toxins), dosage and the diversity of gut microflora in different insect species. The diversity of gut microflora varies across host plants on which the insect feeds and the geographical region from where the insects have been collected (Parmasiva, I., Unpublished). The *H. armigera* populations collected from different host plants and from different regions also exhibit significant differences in their susceptibility to *Bt* toxins. In the absence of gut microbes (when the larvae were fed on diets with antibiotics for three successive generation), the *H. armigera* larval mortality due to *Bt* toxins was drastically reduced as compared to the larvae fed on diets without antibiotics (with gut microbes). Therefore, there is need to study the interaction of antibiotics with *Bt* toxins, the effect of antibiotics on diversity of gut microflora in insects fed on different host plants and from different regions, identify the microbial species that influence the toxicity of *Bt* toxins, the enzymes produced, and their role in potentiation of *Bt* toxins (pro-toxin – toxin conversion) and degradation in the insect mid gut. This information will be important to develop strategies for resistance management in *Bt*-transgenic crops for sustainable crop production and food security.

## Conclusions

The toxicity of commercial *Bt* formulation and the toxin proteins Cry1Ab and Cry1Ac to *H. armigera* larvae was reduced significantly when fed on diets amended with antibiotics. Mortality of insects fed with *Bt* toxins was inversely proportional to the antibiotic concentration. There was no selection for resistance to *Bt* toxins in the absence of gut microflora, suggesting that enteric bacteria will play a major role in evolution of insect populations with resistance to *Bt* toxins/*Bt*-transgenic plants. It will be important to know which of these bacterial species are essential for insecticidal activity/detoxification of *Bt* toxins.

## Methods

The larvae of *H. armigera* were obtained from the laboratory culture maintained at the International Crops Research Institute for the Semi-Arid Tropics (ICRISAT), Patancheru, Andhra Pradesh, India. Larvae were reared on chickpea based artificial diet [35] at 27 ± 1°C, and 12 h photoperiod. The neonates were reared for 5 days in groups of 200 to 250 in 200 ml plastic cups containing a 2 to 3 mm layer of artificial diet on the bottom and sides of the cup. Thereafter, the larvae were transferred individually to six cell-well plates (each cell-well 3.5 cm in diameter, 2 cm in depth) to avoid cannibalism. Adults were provided with 10% sucrose or honey solution on a cotton swab for feeding. Diaper liners, which have a rough surface, were provided as a substrate for egg laying. The liners were removed daily, and the eggs sterilized in 2% sodium hypochlorite solution. The liners with eggs were dried under a table fan and then placed inside the plastic cups with artificial diet.

### Effect of different concentrations of antibiotics on toxicity of *Bt* toxins towards *H. armigera* larvae

The role of gut microflora on the biological activity of *Bt* formulation Biolep®, and pure Cry1Ab and Cry1Ac toxins towards *H. armigera* was studied by rearing the larvae on artificial diet with a range of concentrations (0, 1.95, 3.91, 7.82, 15.63, 31.25, 62.5, 125, 250, and 500 μg ml^−1^ diet) of the antibiotic compounds (gentamicin, penicillin, rifampicin, and streptomycin) (Sigma Chemicals, USA). The larvae were reared on artificial diets amended with antibiotics up to second-instar. Upon moulting to the third-instar, the larvae were transferred to sterile artificial diet without antibiotics, but with ED_50_, LC_50_, and LC_90_ levels of *Bt* formulation (0.005, 0.15, and 0.95%, respectively), Cry1Ab (4, 12, and 35 μg ml^−1^ diet, respectively), and Cry1Ac (4, 12, and 35 μg ml^−1^ diet, respectively) to *H. armigera*. Data were recorded on larval mortality and larval weights five days after rearing the insects on the diets amended with *Bt,* Cry1Ab, or Cry1Ac, corresponding to 10 - day old larvae on the standard artificial diet.

### Effect of antibiotics on toxicity of *Bt*-formulation and toxins Cry1Ab and Cry1Ac towards *H. armigera* larvae across three generations

Role of gut microflora in susceptibility of *H. armigera* to *B. thuringiensis* was studied for three generations by rearing the insects on artificial diet amended with antibiotics (gentamicin, penicillin, rifampicin, and streptomycin, 250 μg each antibiotic ml^−1^ diet). In another treatment, the larvae were reared on artificial diet without antibiotics up to second-instar. Upon moulting to third-instar, the larvae were fed on artificial diet amended with LC_50_ levels of *Bt* formulation (0.15%), Cry1Ab (12 μg ml^−1^ diet), and Cry1Ac (12 μg ml^−1^ diet) to *H. armigera*. Mortality and weights of surviving larvae were recorded 5 days after initiation of experiment. Same protocol was followed for F_2_ and F_3_ generations.

### Data analysis

The data were subjected to analysis of variance. The significance of differences between the treatments was judged by F-test, while the treatment means were compared by least significant difference at P ≤ 0.05. The data on larval mortality across different antibiotic concentrations was subjected to Probit analysis to compute dosage – response relationship (regression equation).

## Competing interests

We have no competing interests, and no organization will in any way gain or lose financially from the publication of this manuscript, either now or in the future. We neither hold nor applying for any patents relating to this study, and have not received reimbursements, fees, funding, or salary from an organization that holds or has applied for patents relating to the content of the manuscript. We have no non-financial competing interests (political, personal, religious, ideological, academic, intellectual, commercial or any other) to declare in relation to this manuscript.

## Authors’ contributions

Dr IP carried out the experiments, recorded and analysed the data, and wrote the first draft of the manuscript. Dr HCS conceived the study, acquisition of funds, and participated in conducting the experiments, interpreted the data and prepared the final draft of the manuscript. Dr PVK acted as PhD supervisor of the student, and read the manuscript. All authors read and approved the final manuscript.
